# STW1 and Its Versatile Pharmacological and Clinical Effects in Rheumatic Disorders: A Comprehensive Report

**DOI:** 10.1155/2020/7841748

**Published:** 2020-07-14

**Authors:** Karl-Josef Gundermann, Jürgen Müller, Karin Kraft

**Affiliations:** ^1^Department of Experimental and Clinical Pharmacology, Pomeranian Medical University, Szczecin 70-111, Poland; ^2^I&D Phytomedicines, Steigerwald Arzneimittelwerk GmbH, Darmstadt 64295, Germany; ^3^Chair of Naturopathic Medicine, University Medicine Rostock, Rostock 18057, Germany

## Abstract

**Aim:**

To review the published and unpublished experimental and clinical studies about the efficacy and tolerability of STW1 and to compare the results to the efficacy and tolerability of investigated NSAIDs in parallel. *Content.* STW1 (Phytodolor®) contains a fixed combination of extracts from aspen leaves and bark (*Populus tremula*), common ash bark (*Fraxinus excelsior*), and goldenrod herb (*Solidago virgaurea*). It belongs to the group of anti-inflammatory and antirheumatic drugs, and it is authorized for the treatment of painful disorders of degenerative and inflammatory rheumatic diseases. The individual components have complementary effects. Its multifocal mode of action includes antiphlogistic, analgesic, antiexudative, antioxidative, antipyretic, and antiproliferative properties. The effects of both STW1 and its components have been verified in comprehensive pharmacological investigations. Open and randomized, placebo- and verum-controlled, and single-blind (sb) or double-blind (db) clinical trials, performed in different subtypes of rheumatic diseases confirm the pharmacological evidence. Its efficacy is comparable to a range of standard nonsteroidal anti-inflammatory drugs (NSAIDs) studied in parallel, but it has a superior safety profile.

**Conclusion:**

STW1 is a reasonable alternative to NSAIDs with comparable efficacy and a superior safety profile. It is also suitable to reduce the intake of NSAIDs.

## 1. Introduction

Many recent advances have been made in the scientific background and therapy of rheumatic diseases [1], but the exact etiology of most of these disorders is still unknown. Autoimmune processes, inflammation, and prooxidant/antioxidant imbalances are often associated with rheumatism causing pain, swelling, and edema. Currently, the pharmacological treatment includes NSAIDs, disease-modifying antirheumatic drugs, biologicals, and glucocorticoids. However, not all patients respond to therapy, and the efficacy of treatment varies with the characteristics of the patients including their genetic background and immune cell phenotype [2]. Furthermore, drugs such as the newer biologicals are not sufficiently investigated for long-term or lifelong routine clinical use, especially with respect to their adverse effect profile. Furthermore, they are quite expensive. As a consequence, complementary medical drugs are widely used by patients with rheumatic diseases. This has many potential implications in a group of predominantly elderly patients with altered pharmacokinetics, comorbidities, and polypharmacy of potentially toxic drugs [3, 4].

As a broad range of subjective symptoms, clinical findings, and biochemical changes has to be effectively treated in rheumatology, herbal extracts with their high number of constituents are considered as a major component of complementary medical therapies. However, there are only few well-investigated herbal medical drugs with standardized composition available for the treatment of rheumatic diseases. One of them is STW1 (Phytodolor®, Steigerwald GmbH, Darmstadt, Germany) [5]. The standardized herbal combination contains 60 mL extract of fresh bark and leaves of *Populus tremula* (drug extract ratio (DER) 4.5 : 1), 20 mL extract of fresh bark of *Fraxinus excelsior* (DER 4.5 : 1), and 20 mL extract of fresh *Solidago virgaurea* (DER 4.8 : 1), each as a 60 vol.% ethanolic extracts. The recommended daily dose is 20 to 30 drops 3-4 times daily (up to 40 drops in severe cases).

The following active and additive properties are attributed to the three extract components in STW1 [6]:

- *P. tremula*: anti-inflammatory, analgesic, antibacterial, and spasmolytic
- *F. excelsior*: analgesic, antioxidative, and antiphlogistic
- *S. virgaurea*: diuretic, analgesic, antibacterial, antiexudative, and mildly spasmolytic

This report summarizes both the published experimental and clinical studies and all the unpublished ones, which were performed for registration purposes, and it compares the results to the efficacy and tolerability of investigated NSAIDs in parallel.

## 2. Methods

Database research was done using Cochrane, EMBASE, PubMed, other published review articles, meta-analyses (e.g., [5–8]), and internal research reports of the company Steigerwald Arzneimittelwerk, Darmstadt, Germany. The latter ones were conducted by the manufacturer for product development and testing and for product registrations.

Twelve publications and abstracts related to *in vitro* studies investigated the antioxidative/anti-inflammatory effects of STW1 in 14 different models.  Table 1 compares the effects of STW1 to the 3 single herbal extracts. Nine *in situ/in vivo* studies determined the effects of STW1 and its individual herbal extracts in different models of inflammation, edema, pain, and fever (Table 2). Twenty-three open comparative and noncomparative clinical studies were completed by 18, only partly published single- and double-blind clinical studies (Table 3).

## 3. Results

### 3.1. In Vitro Studies

The *in vitro* studies were carried out to obtain explanatory insights into the mode of action and into the extent of the anti-inflammatory properties of STW1, especially with respect to its antioxidative properties. Fundamentally, they can be divided into three categories:

- Studies on simple biochemical systems (photodynamic excitation reactions driven by rose bengal and riboflavin, peroxynitrite system, Fenton/Haber–Weiss system, dihydrofolate reductase (DHFR) system in the presence of copper ions, and 2,2'-azobis (2-amidinopropane) dihydrochloride (AAPH) system)
- Studies on enzyme systems (myeloperoxidase (MPO) reaction, xanthine oxidase (XOD) system, reduced nicotinamide adenine dinucleotide phosphate (NADPH) oxidase/diaphorase, and lipoxygenase reactions)
- Studies on complex model reactions (MPO/elastase/*α*1 antiproteinase system, diene conjugation of low-density lipoprotein (LDL) particles, tyrosine nitration by peroxidase nitrite, formation of radicals in neutrophilic granulocytes, and interferon-gamma/lipopolysaccharide- (IFN-*γ*/LPS-) stimulated apoptosis of monocytes).

Table 1 summarizes the *in vitro* results, in which all three herbal extracts were tested in addition to STW1.

STW1 showed potential for scavenging radical oxygen species (ROS) in different systems, which are relevant for the formation of ROS *in vivo* in inflammatory sites: rose bengal or riboflavin, XOD, diaphorase, and lipoxygenase, and it blocked both the peroxynitrite-dependent nitration and the enzyme- (peroxidase-) catalyzed reaction [8, 9].

STW1, *P. tremula*, or *F. excelsior* inhibited MPO-catalyzed reactions in different MPO assays (H_2_O_2_/MPO; X/XOD/MPO; activated granulocytes; elastase/*α*1-PI/MPO), whereas *S. virgaurea* showed no or little effect [10].

While basal radical production of leukocytes was only slightly influenced by STW1 and its extracts, strong inhibiting effects were observed after activation with zymosan, STW1 being more active than its single extracts (synergistic/supra-additive mode of action) [11].

All extracts showed a radical scavenging effect in the AAPH reaction; the extract of *F. excelsior* was the strongest, and the effect of the combination was additive [11, 12].

The results are completed by investigations with STW1 versus two salix extracts on copper-catalyzed oxidative destructions and on superoxide-dependent and superoxide-independent nitrite formation from hydroxylamine [40–42]. LDL oxidation by copper ions was strongly inhibited by both extracts and STW1 in a concentration range of 4 to 7 *µ*g/mL. Likewise, ethene release from 2-keto-5-methylthiobutyrate (KMB) was strongly inhibited in a reaction driven by dihydroxyfumarate in the presence of copper ions [40, 41]. Furthermore, the radical scavenging activities of STW1 and other extracts were demonstrated by inhibiting ethene release from KMB induced by Fenton-type oxidants and by the inhibition of the formation of nitrogen monoxide (measurable as nitrite formation) from hydroxylamine including XOD in the presence or absence of myoglobin [42].

Synchronized human fibroblasts not stimulated or activated with LPS were treated with STW1 and its components to investigate the gene and protein expression profile of genes involved in immunoregulation, inflammation, and apoptosis. Each of the single extracts modulated a different number of genes based on a microarray assessment. Under LPS activation, *P. tremula* modulated 51 genes, *F. excelsior* 31 genes, and *S. virgaurea* 24 genes. The extract combination modulated 40 genes, demonstrating that the number of active components in an extract does not necessarily determine the number of targets and also that the gene expression profiles of the single extracts do not allow a prediction of the gene expression profiles of their combination. STW1 reduced the proinflammatory cytokines interleukin-13 (IL-13) and tumor necrosis factor-alpha (TNF-*α*), and the gene expression of the proinflammatory IL-6 and IL-8 as chemokine of the immune system, monocyte chemoattractant protein 1 (MCP-1), and growth-regulated oncogene alpha (Gro-*α*). The genes regulated by STW1 and its components showed an overlap of 57.9%. STW1 had the maximum individual overlap with *P. tremula* (36.5%) and an even greater overlap with acetylsalicylic acid (ASA; 52.9%) [13].

The results on cyto- and chemokines were completed by further ones [14]: the influence of each extract on an inflammatory cytokine and chemokine network (CCN) was confirmed to be specific. The response to STW1 could not be predicted from the network of the three plant extracts. This was the case both in the presence or absence of LPS and at the level of protein and gene expression. Salicylate-based herbal drugs, such as STW1, provoke pro- and anti-inflammatory CCN responses under nonstress conditions, which adapt to anti-inflammatory responses after LPS stimulation [14].

The activity of DHFR, which is connected with rapidly proliferating cells with proinflammatory activity, such as bacteria, was significantly inhibited by STW1 and its three herbal extracts [15].

The extract combination has also been shown to inhibit the proinflammatory TNF-*α* gene expression and the synthesis of the TNF-*α* and COX-2 proteins in IFN-*γ*/LPS-stimulated human monocytes. In addition, STW1 significantly inhibited the proinflammatory reduced apoptosis rate. These anti-inflammatory effects were comparable to those of diclofenac [16].

It can be concluded from these *in vitro* investigations that STW1 has potent radical scavenging and anti-inflammatory properties. Comparing semiquantitatively all studies, in which STW1 was tested against its three herbal extracts (Table 1), STW1 and *P. tremula* seem to be more active than *F. excelsior* and *S. virgaurea*.

### 3.2. In Vivo Studies

The results from the *in vitro* models on the anti-inflammatory and antioxidative properties of STW1 were confirmed and completed by *in vivo* investigations (Table 2).

STW1 decreased local tissue hormones. It inhibited the formation of the lipoxygenase product leukotriene B_4_ (LTB_4_) from proinflammatory neutrophilic leukocytes. This finding was confirmed *in situ* on the perfused rabbit ear and sensitized perfused guinea pig lung, respectively. STW1 significantly inhibited the synthesis of the prostaglandins PGE_2_, PGI_2_, and PGD_2_, comparable to the effects of indomethacin. The results were also comparable to the release of histamine, prostaglandins, and leukotrienes in the sensitized, perfused guinea pig lung model. The inhibition of PGE_2_ was stronger than that of PGI_2_ and consequently less incriminating for the microcirculation in the gastric mucosal membrane than that observed for NSAIDs.

STW1 significantly reduced acute inflammation in the carrageenan- or dextran-induced rat paw edema comparable to 3.0 mg of diclofenac. The fixed combination of STW1 was more effective than the single extracts. Moreover, Freund's adjuvant-induced arthritis was significantly reduced, and the volume increase was inhibited.

The analgesic effects of STW1 in the phenylquinone writhing test, a deep pain model, and the Randall–Selitto paw pressure test seem to be based on an inhibition of the synthesis of inflammatory and pain mediators in the peripheral inflammatory tissue. In contrast to the *in vitro* results with the DHFR activity model, the inhibition of granuloma formation in the cotton pellet test, a model for chronic proliferative inflammation, was stronger with STW1 than with the single herbal extracts, but not significant versus control, while the antiproliferative effect in adjuvant-induced arthritis was significantly superior to control. Additionally, an antipyretic effect in brewer's yeast-induced hyperthermia was observed for STW1 and its single extracts. STW1 had a significant and rapid antipyretic activity, which was less expressed than that of ASA but more distinct than that of the single extracts.

In conclusion, the single extracts of *S. virgaurea*, *P. tremula*, and *F. excelsior* are active as antioxidants and anti-inflammatory agents, but the fixed combination is more effective. *S. virgaurea* shows the lowest level of inhibition (Tables 1 and 2).

### 3.3. Clinical Studies

#### 3.3.1. Randomized Controlled Studies: Efficacy

Tables 3 and 4 summarize the design and investigated parameters of the performed randomized single-blind (sb) and double-blind (db) studies. Nine db trials were carried out versus placebo. In four of these studies, patients were permitted to take additionally up to 6 × 25 mg diclofenac per day for pain control. Seven further db and sb studies tested the efficacy of STW1 against 3 × 25 mg diclofenac, in 3 studies up to 6 × 25 mg diclofenac per day. One sb study and the open phase of db study compared STW1 to 75 to 150 mg indomethacin daily and one db study to 1 × 20 mg piroxicam per day. Two db studies used *Populus* extract as the reference product, and one sb study tested STW1 with iontophoresis versus iontophoresis with sodium chloride. The placebo-controlled, db studies comprised degenerative rheumatic diseases, such as osteoarthrosis, osteoarthritis, and epicondylitis lateralis, as well as inflammatory diseases, such as rheumatoid arthritis. In the case of the db and sb studies against NSAIDs or versus *Populus e*xtract, a broader indication field was investigated including stage II and III rheumatoid arthritis, gonarthrosis and coxarthrosis, cervical spine syndrome/lumbar spine syndromes, lateral epicondylitis, shoulder-arm syndrome, and periarticular fibrositis.

Three out of the four randomized db studies performed with *STW1* versus *placebo and permitted comedication* showed positive results for STW1 [18–22]. On average, the drug enabled a constantly lower intake of diclofenac with or without paracetamol. The latest one of that category was carried out by Huber [21, 22]. The patients suffering from degenerative rheumatic diseases took 3 × 30 drops of STW1 for three weeks, or a corresponding placebo. Under STW1, the 18 patients took additionally a total of 100 mg diclofenac and one 500 mg tablet of paracetamol, whereas the 20 placebo patients took 2,400 mg diclofenac and three 500 mg tablets of paracetamol. Calculated over days, the STW1 group required comedication on three days and the placebo group required comedication on 47 days. The clinical improvements were comparable.

The aim of the five randomized db studies with *STW1 versus placebo without comedication* was to prove the efficacy of the drug especially for its pain and mobility effect beyond placebo. Three studies showed significant advantages for STW1 [23, 24, 26, 29, 30], and two studies showed a positive trend in favor of it [25, 27]. For example, Schreckenberger [28–30] combined a placebo-controlled double-blind trial with one single-blind study against diclofenac to evaluate the superiority of the test drug versus placebo in epicondylitis lateralis and the equipotency between STW1 and diclofenac at a dose of 3 × 40 drops/d or 3 × 25 mg diclofenac/d, respectively. The study was carried out for two weeks with 15 patients per group.

STW1 achieved the best results with respect to the decreased pain intensity and increased strength of the patients. In the latter parameter, the group difference was statistically significant (*P* < 0.001). The STW1 group was the only one without observed adverse effects. A second predominant study was that one from Bernhardt et al. [26] who included patients suffering exclusively from pain due to degenerative rheumatic diseases. Patients with stage II moderate osteoarthritis, as defined by the “American Rheumatism Association” (ARA) (gonarthritis and coxarthritis, and cervical and lumbar spine syndromes), were selected for therapy. The two groups of the db study with 36/36 patients were compared to an open field group of the same size. The result of this carefully designed study demonstrated equivalent efficacy between STW1 at 3 × 30 drops/d and piroxicam at a dose of 1 × 20 mg-tablet/d and better tolerability in favour of STW1. A distinct superiority compared to the placebo group was demonstrated especially for the most intense parameter “motor pain” as well as for the final assessment by the physician and the patient. Changes in finger-to-floor distance and grasping strength by STW1 and piroxicam (32.7% and 32.2%) differed significantly from placebo, too (*P* < 0.05).

Eight comparative studies with *STW1 versus NSAIDs with and without permitted comedication* were performed. Baumann et al. [32] were the first ones using the double-dummy method with STW1. A total of 52 patients with activated gonarthrosis, coxarthrosis, or shoulder-arm syndrome took STW1 for two weeks at a dosage of 3 × 30 to 3 × 40 drops/d, and 56 patients took 3 × 25 mg diclofenac/d, in each case combined with the corresponding placebo. A distinct improvement in the symptoms and a diminution in the pathologically elevated BSR as a nonspecific inflammatory parameter were observed in both groups. The efficacy of STW1 was comparable to that of diclofenac. The two preparations had a greater effect on gonarthrosis and shoulder-arm syndrome than on coxarthrosis. A larger multicentre, double-dummy, db study versus diclofenac followed [33] two hundred seventy-seven patients with osteoarthritis received STW1 at a dose of 3 × 40 drops/d, and 140 patients were treated with 3 × 25 mg diclofenac/d. During the 4-week therapy, there was no significant difference in the efficacy of the two preparations with respect to short-term pain reduction. The mean pain score dropped significantly (*P* < 0.01) in both groups, and the intake of paracetamol was comparable.

In conclusion, the controlled clinical trials showed superior efficacy of STW1 versus placebo and comparable efficacy to NSAIDs in moderate doses.

#### 3.3.2. Further Clinical Studies

Twenty-one additional open, noncomparative studies, carried out for >2 to 72 weeks [43], served either to define the indications [44, 45], were pilot studies for the following sb and db studies [46, 47], or tested special hypotheses, such as the reduction of the use of glucocorticoids [48], the effect of STW1 on Bechterew's disease [49], or on therapy with sulfonylurea in diabetes mellitus [50]. The other ones were therapeutic reports [51, 52], observational studies to record adverse drug effects [53–55], and a retrolective study, performed to document the long-term treatment effectiveness of STW1 [56]. The indications of the open studies were especially painful conditions after orthopedic operations, lumbago and ischialgia, fibromyalgia, periarthritis, Bechterew's disease, pediatric inflammatory joint diseases, and chronic bone conditions without clear organic cause.

The key variables of the retrolective study [56] with 300 patients were general health, pressure pain of joints, restriction of motion, and swollen joints, supported by the variables walking distance, handgrip force, stair climbing, and C-reactive protein (CRP). During the 72-week treatment with STW1, subjective, objective, and laboratory variables were continuously improved. Pretreatment with mainly ibuprofen did not significantly influence the therapeutic success with STW1. These cases also showed a permanent improvement in the clinical course of the disease. Side effects or interactions were not reported.

A comprehensive observational study was carried out by general practitioners and internists in private practice [53]. A total of 1,827 patients with degenerative rheumatic diseases (68%), inflammatory diseases (20%), or “mixed forms” (12%) were treated for four weeks with 3 × 30 drops of STW1 per day. Of these patients, 1,068 (58%) did not take any further analgesic or antirheumatic drug during therapy. The effect was assessed as being positive in 73.8% of the patients. Also the physician's global assessment of the tolerability of STW1 was positive for 96.9% of the patients. Only 3% of the patients discontinued the therapy. 16% of the patients reported adverse effects (mainly abdominal pain, flatulence, and vertigo), and the incidence of the adverse effects was markedly lower compared to the prestudy medication.

#### 3.3.3. Meta-Analyses and Systematic Reviews

In a first systematic review from 1999, it was concluded that STW1 is a safe and effective treatment for musculoskeletal pain [57]. A second review deduced from the available data that STW1 relieved many osteoarthritic symptoms, particularly pain, in a reasonably large number of RCTs of good methodological quality. According to the authors, the trials demonstrated significant results for pain reduction, mobility, and NSAID consumption, and they also suggested that STW1 is as effective as NSAIDs, but has fewer adverse effects [58]. A meta-analysis from the same year included four randomized, placebo-controlled, db studies [23–27] with the main outcome variables pain on movement, enduring, or at rest. Clear superiority of STW1 was evident versus placebo during a treatment period of 3-4 weeks [59]. As this meta-analysis was not published, a Cochrane database of systematic reviews on herbal therapy for rheumatoid arthritis could not conclusively prove the efficacy of STW1, as only published studies were included [6]. However, a subsequently performed meta-analysis of eleven randomised controlled trials in patients with musculoskeletal disorders provided supporting evidence of effective pain reduction at rest and during motion. Furthermore, STW1 was significantly superior compared to placebo in patients' global assessment of efficacy and in the subpopulation with “predominantly other rheumatic diseases”, but not in the subpopulation with “predominantly gonarthrosis”. It did not differ significantly in efficacy from NSAIDs, neither in the entire population nor in the subpopulations. Only minor adverse events (AEs), such as gastrointestinal complaints, were reported (placebo 8.1%; STW1 14.2%; NSAIDs 18.9%) [60].

A further meta-analysis from 2018 analysed the efficacy of STW1 versus NSAIDs in the treatment of rheumatic symptoms [61]. Six at least sb studies with a total of 712 patients were included. Four studies compared STW1 to diclofenac, and one study each to ibuprofen or piroxicam. Various pain parameters and limitation of mobility were evaluated. Patients should have been treated for at least two weeks. The results per parameter were summarized as follows: (a) for pain in general, all results were in favor of STW1 with an estimated difference of 0.28 points of a score from zero (no pain) to four (severe pain) (*P* < 0.05), but the small sample size limited the validity of the results; (b) for pain at rest and pain during motion, no difference was seen between STW1 and NSAIDs. The results of all studies showed a high degree of consistency and homogeneity, but the crude pooling did not provide valid results due to different treatment allocation ratios in the studies; (c) for pressure pain, one study result was in favor of NSAID and one in favor of STW1, which prevented a meta-analysis due to high heterogeneity and did not allow to combine the results; (d) for endurance pain, the results were ambiguous. One study did not show a difference between the two treatment groups, while the other one showed an advantage for the NSAID. However, as the baseline values were different between the treatments with a lower symptom mean score for STW1, no clear interpretation was possible; (e) for limitations of mobility, one study showed no difference between the groups while the other two studies favored either the NSAID or STW1. Nevertheless, no meaningful interpretation was possible due to substantial difference in the baseline values. In conclusion, the improvements of the investigated symptoms were comparable for STW1 and NSAIDs.

### 3.4. Safety and Compatibility

#### 3.4.1. Preclinical Studies

STW1 caused no relevant effects in extended safety pharmacological studies in mice, rats, guinea pigs, rabbits, and beagle dogs [5]. Due to lack of toxicity, LD_50_ values were not technically achievable for rodents in acute and subacute toxicity investigations. For beagles, subacute toxicity studies were performed. No toxicological effects were found in two reproductivity studies with a daily oral dosage corresponding to 10 mL/kg b.w., including no effects on the growth of the animals, the mating behavior, the fertility, or litter size. Furthermore, STW1 did not exert any mutagenic effects in two *in vivo* tests (mammalian spot test and micronucleus test in bone marrow cells of mice) and in one *in vivo/in vitro* test (unscheduled DNA test on rat hepatocytes) [62].

#### 3.4.2. Clinical Studies

Clinically, no serious AEs were reported [60], and the hierarchy of incidence of spontaneously reported nonserious AE was placebo <STW1 <NSAIDs. The most common AEs were with both STW1 and NSAID gastrointestinal complaints (e.g., epigastric symptoms), followed by unspecified symptoms, such as headache, vertigo, and skin disorders (e.g., exanthema). However, AE prompted only few patients to withdraw from the trials. Overall, the frequencies of AE and withdrawals were similar to those observed in the abovementioned observational study with 1,827 patients (15.6% reported spontaneously AE, and 3.2% withdrew) [53].

Besides desired effects, such as reduced blood sedimentation rate (BSR) and CRP values, the laboratory analyses did not show abnormal findings [53].

## 4. Discussion

Currently, treatments targeting cytokines, including TNF-*α* antibodies, anti-IL-6 receptor antibodies, and IL-1 receptor antagonists, are frequently used for rheumatic inflammatory diseases additionally to antiphlogistic and disease-modifying antirheumatic drugs (DMARDs). Although advances have been made with DMARDs and biological agents, symptoms, such as pain, remain insufficiently controlled for many patients. Often, it takes several weeks until these drugs become effective, which on top of corticosteroids requests additional pain treatment. Moreover, their clinical use remains limited due to their adverse effects and complications, such as addiction, increased rate of infections, and gastrointestinal bleedings, and some of them are very expensive. Therefore, though herbal medicinal drugs do not seem to act faster than the DMARDs, they are frequently considered to be useful as adjuvant therapy for this condition. They offer a versatile approach to treat the multidimensional nature of symptoms in osteoarthritis, rheumatic diseases, and musculoskeletal complaints, out of which chronic pain is the leading indication for their use. They are especially valuable for geriatric patients, as they reduce pain, improve quality of life, maintain autonomy, avoid need of care, are well tolerated, and reduce taking of drugs with serious side effects.

STW1 is one of few well-investigated herbal medicinal drugs for the treatment of painful disorders of degenerative and inflammatory rheumatic diseases. In total, 4,332 patients were evaluated in 41 studies, out of these 3,517 patients were treated with STW1. With the exception of one study [50], the purpose was to investigate the influence of the drug on painful inflammatory or degenerative rheumatic diseases. Pain, mobility, swollen joints, systemic inflammation (as measured by BSR and CRP), and saving of additional NSAIDs were the most frequently measured variables. STW1 was shown to be not only active on symptoms and clinical findings but also (*in vitro*) on proinflammatory cytokines and chemokines [14, 16]. Eleven clinical trials and one retrolective study demonstrated that the benefit-risk profile of STW1 was not changed after six to 72 weeks of administration [18, 31, 39, 49, 54–56, 63–67]. Conclusions from systematic reviews of STW1 range from “potential (effective) in alleviating pain” [68] or “suggested reduced pain” [69] or “moderate support for pain” [70] to “significant pain reduction” [58]. Meta-analyses and systematic reviews confirm the value of STW1 as anti-inflammatory and antirheumatic medicine and for the treatment of painful disorders of degenerative and inflammatory rheumatic diseases.

**Figure 1:**
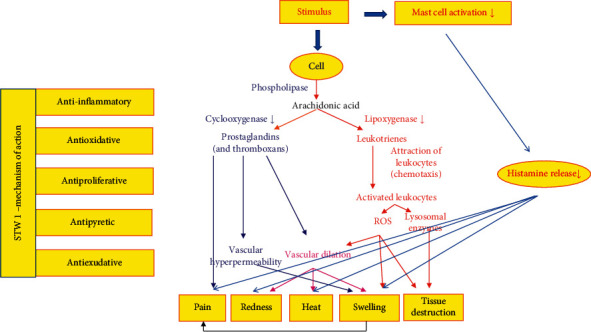
Mechanism of action of STW1 in correlation with subjective and clinical findings.

Figure 1 summarizes the pharmacological effects and correlates the mechanism of action of STW 1 with the observed subjective and clinical improvements. It is postulated that STW1 especially by decreasing proinflammatory prostaglandins and leukotrienes leads to diminished amount of ROS and lysosomal enzymes, as well as to reduced vascular hyperpermeability and dilation. As a consequence, pain, redness, heat, swelling, and loss of function decrease.

Relying on these results, a cost-saving evaluation was done in Australia [71]. The analysis compared STW1 to diclofenac assuming the efficacy and health outcomes of each treatment being equivalent in the treatment of osteoarthritis. The analysis revealed that the treatment of osteoarthritis was cost reducing for people using STW1 rather than diclofenac, with around a 24% price premium estimated.

A multitude of the conducted studies with STW1 was not published. This comprehensive report is based on studies mentioned in the pharmacological-toxicological and clinical expert reports from 2012, which were used for registration purpose [43, 62]. As most of the sb and db studies were performed at the end of the eighties and beginning of the nineties, a higher level of evidence than that of the currently existing studies is recommended with well-designed, fully powered, confirmatory clinical trials. Additionally, as rheumatic diseases are of complex nature, studies are recommended, which combine STW1 with other antirheumatic drugs, especially DMARDs and biologicals.

## 5. Summary and Conclusion

The presently available pharmacological and clinical results show a significant and low to moderate efficacy of STW1 especially in pain reduction in patients suffering from rheumatic disorders and diseases, though different evaluation methods were used.

The clinically relevant positive changes observed with regard to pain and motion variables in inflammatory, degenerative, and soft tissue rheumatism as well as for fibromyalgia, combined with a good tolerability, support the administration of STW1 as an antirheumatic agent, especially for patients with mild to moderate pain in the context of degenerative rheumatic disease. The effects are comparable with the NSAIDs studied in parallel, such as diclofenac, indomethacin, and piroxicam. The improvement continues over time as has been shown by long-term treatments. Meta-analyses validate the results from the sb and db studies. The safety and tolerability of STW1 are near to placebo and superior to the investigated NSAIDs, which enables STW1 to substitute or reduce the application of NSAIDs.

## Figures and Tables

**Table 1:** Effects of STW1 in comparison with its individual herbal extracts in different *in vitro* models for antioxidative/anti-inflammatory effects (semiquantitative assessment)^*∗*^.

Models	Rose bengal	Riboflavin	Xanthine oxidase (XOD)	Diaphorase	Lipoxygenase	Myeloperoxidase (MPO)	Zymosan-activated leukocytes	AAPH decomposition^*∗∗*^	Gene and protein expression in fibroblasts^*∗∗∗*^	Cytokine and chemokine network responses in fibroblasts^*∗∗∗*^	DHFR^*∗∗∗∗*^	IFN-*γ*/LPS-activated human monocytes ^*∗∗∗∗∗*^	Total x
Properties	Antioxidative								Anti-inflammatory				

References	[8, 9]					[10]	[11]	[11, 12]	[13]	[14]	[15]	[16]	
STW1	x	xx	x	x	xx	x	xx	xx	xx	Nonadditive cytokine and chemokine network profiles	xx	xx	18
*Populus tremula*	x	xx	xx	x	xx	x	xx	xx	xx		x	xx	18
*Fraxinus excelsior*	x	x	xx	x	x	x	x	xx	x		xx	x	14
*Solidago virgaurea*	x	x	xx	x	x	O	x	x	x		x	(x)	10.5

^*∗*^Only those studies and models are listed, in which in addition to STW1 all three herbal extracts were tested. ^*∗∗*^2,2'-Azobis (2-amidinopropane) dihydrochloride. ^*∗∗∗*^In the presence or absence of LPS. ^*∗∗∗∗*^Dihydrofolate reductase. ^*∗∗∗∗∗*^IFN-*γ*/LPS = interferon-*γ*/lipopolysaccharide; xx = strongest effect; xx = strong effect; x = medium effect; (x) = slight effect; O = no effect.

**Table 2:** Effects of STW1 and its individual herbal extracts in different *in situ/in vivo* models of inflammation, edema, pain, and fever (semiquantitative assessment)^*∗*^.

Models	Phenylquinonewrithing test (analgesic effect)	Carrageenan-induced paw edema (anti-inflammatory/antiexudative effect)	Adjuvant-induced arthritis (antiproliferative/anti-inflammatory effect)	Dextran rat paw edema (antiexudative/anti-inflammatory effect)	Inhibition of the generation of inflammatory mediators^*∗∗*^						Brewer's yeast-induced pyrexia (ant-pyretic/analgesic effect)	Cotton pellet test^*∗∗*^ (antiproliferative/anti-inflammatory effect)	Total x
					Ear	Lung			Granulocytes				
					PGs	HIST	PGs	LTs	LTB_4_	5-HETE			
References	RR 03/84, 01/85, and 24/89	RR 04/85, 15/87, and 26/89 [8]	RR 04/85, 15/87, and 26/89 [8]	RR 13/87 and 14/87 [8]	RR03/89 and 20/89	RR 7/90 and 17/91			RR 23/89		RR 04/86, 12/87, 21/88, 27/89, 03/90, and 04/90	RR 05/85	
STW1	x	xx	xx	xx	x	X	xx	x	x	x	Xx	xx	18
*Populus tr.*	xx	x	(x)	x	xx	xx	(x)	xx	xx	x	X	(x)	15.5
*Fraxinus exc*.	x	x	x	x	x	(x)	x	xx	(x)	(x)	X	x	11.5
*Solidago vir.*	x	x	x	x	o	xx	x	(x)	x	xx	X	(x)	12

^*∗*^Effects of aqueous-alcoholic and of dry extracts. ^*∗∗*^Effects of aqueous-alcoholic extracts. HIST = histamine; PGs = prostaglandins; LTs = leukotrienes; LTB_4_ = leukotriene B_4_; 5-HETE = 5-hydroxy-eicosatetraenoic acid; RR = research report; xx = strongest effect; xx = strong effect; x = medium effect; (x) = slight effect; O = no effect.

**Table 3:** Study design of randomized single- and double-blind studies with STW1.

Primary investigator [ref]	Indication	Study design	Comparison groups	Number of patients	Dose (drops/day)	Washout (days)	Duration of therapy	Plus NSAID	Published	Comment
Meier [17]	RA	db	Placebo, “nothing”	5, 5, 5	3 × 30		2 weeks	Diclo.	No	Pilot study
Eberl et al. [18]	RA	db	Placebo	20, 17	3 × 30		1 year	Diclo.	No	
Schadler [19, 20]	OA	db	Placebo	15, 15	3 × 40		2 × 7 days	Diclo.	Yes	Crossover
Huber [21, 22]	MD	db	Placebo	18, 20	3 × 30	1	3 weeks	Diclo.	Yes	
Hahn and Hübner-Steiner [23, 24]	MD	db, open	Placebo, indomethacin	15, 15, 15	3 × 30 (40)		4 weeks		Yes	
Speck et al. [25]	MD	db	Placebo	15, 11, 11, 10	3 × 30, dd, hd		4 weeks		No	Various concentrations
Bernhardt et al. [26]	MD	db, open	Placebo, piroxicam	36, 36, 36	3 × 30		4 weeks		No	
Müller-Faßbender [27]	OA	db	Placebo, *Populus* extract	71, 72, 72	3 × 40	2	3 weeks		No	
Schreckenberger [28]	Epicondylitis	sb	Diclofenac	16, 15	3 × 30		1 week		No	
Schreckenberger [29, 30]	Epicondylitis	db, sb	Placebo, diclofenac	15, 15, 15	3 × 40		2 weeks		Yes	
Schadler [19, 20]	OA	sb	Diclofenac	15, 15	3 × 30		3 weeks		Yes	
Schadler and Kalmbach [31]	MD	sb	Diclofenac	10/10	3 × 30 (40)		24 weeks		No	
Baumann et al. [32]	OA	db	Diclofenac	52, 56	3 × 30 (40)		2 weeks		No	Double-dummy
Herzog et al. [33]	MD	db	Diclofenac	277, 140	3 × 40	7	4 weeks	Paracet.	No	Double-dummy
Hawel et al. [34–36]	MD	db	Diclofenac	108, 106	3 × 40		3 weeks		No	Double-dummy
Michael and Sörensen [37]	MD	db	*Populus* extract	12, 13	3 × 30 (40)		4 weeks		No	
Botzenhardt [38]	RA	sb	Indomethacin	16, 15	3 × 30		3 weeks	Paracet.	No	
Vajda and Kiss-Antal [39]	OA	sb	Iontophoresis	15, 15	2 × 5 mL		3 weeks	Paracet.	No	

RA = rheumatoid arthritis; OA = osteoarthritis; MD = various musculoskeletal disorders; dd = double dose; hd = half dose; Diclo. = diclofenac; Paracet. = paracetamol.

**Table 4:** Study parameters of randomized single- and double-blind studies with STW1.

Primary investigator [ref]	MP	CP	MI	MS	T	PExt	PPat	P	SW	RP	FPR	FI	PSM	NP	ATI	Comment
Meier [17]								x	x						+	
Eberl et al. [18]															+	Joint index, duration of morning stiffness
Schadler [19, 20]	x				x					x					+	
Huber [21, 22]		x														
Hahn and Hubner-Steiner [23, 24]	x	x	x													
Speck et al. [25]	x	x		x												
Bernhardt et al. [26]	x	x	x													
Müller-Faßbender [27]	a									a						+ knee joint index
Schreckenberger [28]					x	x										Only sum scores
Schreckenberger [29, 30]					x	x	x									
Schadler [19, 20]			x					x								
Schadler and Kalmbach [31]																Only lab. param.
Baumann et al. [32]	x								x	x	x	x				
Herzog et al. [33]	x									x			x		+	
Hawel et al. [34–36]	x				x					x		x				Including shoulder arm-syndrome
Michael and Sörensen [37]	x	x		x												
Botzenhardt [38]				a												+ strength of grip, sum score of 15 joints
Vajda and Kiss-Antal [39]	a									a	a			a		

MP = motor pain; CP = constant pain; MI = motor impairment, inhibition of active mobility; MS = morning stiffness; T = tenderness; PExt = pain during maximal extension against resistance; PPat = patient's pain assessment; P = pain; SW = swelling; RP = rest pain; FPR = first pain after resting; FI = functional impairment; PSM = pain at start of movement; NP = nocturnal pain; ATI = additional tablet intake; x = ordinal scale; a = visual analogue scale.

## References

[1] Watts R. A., Conaghan P. G., Denton C., Foster H., Isaacs J., Müller-Ladner U. (2018). *Oxford Textbook of Rheumatology*.

[2] Kroenke K., Krebs E. E., Bair M. J. (2009). Pharmacotherapy of chronic pain: a synthesis of recommendations from systematic reviews. *General Hospital Psychiatry*.

[3] Tanaka Y., Hirata S., Saleem B., Emery P. (2013). Discontinuation of biologics in patients with rheumatoid arthritis. *Clinical and Experimental Rheumatology*.

[4] Zhao S., Otieno F., Akpan A., Moots R. J. (2017). Complementary and alternative medicine use in rheumatoid arthritis: considerations for the pharmacological management of elderly patients. *Drugs & Aging*.

[5] Gundermann K.-J., Müller J. (2007). Phytodolor®-effects and efficacy of a herbal medicine. *Wiener Medizinische Wochenschrift*.

[6] Cameron M., Gagnier J. J., Chrubasik S. (2011). Herbal therapy for treating rheumatoid arthritis. *The Cochrane Database of Systematic Reviews*.

[7] Medical Economics Company (2007). *PDR (Physician’s Desk Reference) for Herbal Medicines*.

[8] Meyer B., Schneider W., Elstner E. F. (1995). Antioxidative properties of alcoholic extracts from *Fraxinus excelsior*, *Populus tremula* and *Solidago virgaurea*. *Drug Research*.

[9] Schempp H., Weiser D., Elstner E. F. (2000). Biochemical model reactions indicative of inflammatory processes-activities of extracts from Fraxinus excelsior and Populustremula. *Drug Research*.

[10] von Kruedener S., Schneider W., Elstner E. F. (1996). Effects of extracts from *Populus tremula* L, *Solidago virgaurea* L. and *Fraxinus excelsior* L. on various myeloperoxidase systems. *Drug Research*.

[11] Hartwich I., Germann I., Kelber O., Müller J. (2006). The antirheumaticphytomedicine STW 1 has antioxidative effects. *Journal of Rheumatology*.

[12] Germann I., Kelber O., Müller J., Weiser D. (2005). Radical scavenging properties of the antirheumatic phytomedicine Phytodolor and its components. *Archives of Pharmacology*.

[13] Rohnert U., Schneider W., Elstner E. F. (1998). Superoxide-dependent and -independent nitrite formation from hydroxylamine: inhibition by plant extracts. *Zeitschrift für Naturforschung C*.

[14] Ulrich-Merzenich G., Hartbrod F., Kelber O., Müller J., Koptina A., Zeitler H. (2017). Salicylate-based phytopharmaceuticals induce adaptive cytokine and chemokine network responses in human fibroblast cultures. *Phytomedicine*.

[15] Strehl E., Schneider W., Elstner E. F. (1995). Inhibition of dihydrofolate reductase activity by alcoholic extracts from *Fraxinus excelsior*, *Populus tremula* and *Solidago virgaurea*. *Drug Research*.

[16] Schaser J., Bonaterra G. A., Kelber O., Weiser D D. (2006). Investigation of anti-inflammatory effects of the phytopharmaceutical Phytodolor® and its individual components in a monocyte test model. *Perfusion*.

[17] G. Meier: Phytodolor N versus placebo in rheumatoid arthritis. Research Report of August 1987

[18] R. Eberl, A. Dunky, B. Leeb, A. Wohanka: Savings of non-steroidal antirheumatics by Phytodolor-placebo-controlled, double-blind study over a period of one year per patient. Research Report of 1988

[19] W. Schadler, Phytodolor—double-blind study against placebo. Comparative clinical trial. Research Report of January 1988

[20] Schadler W. (1988). Phytodolor® for the treatment of activated arthrosis. *Journal of Rheumatology*.

[21] B. Huber: On the additional requirement of analgesic medication in the treatment of degenerative rheumatic diseases with Phytodolor®-results of a placebo-controlled, double-blind clinical trial. Research Report of February 1990

[22] Huber B. (1991). Therapy of degenerative rheumatic diseases. Requirement for additional analgesic medication under treatment with Phytodolor® N. *Fortschr Med*.

[23] S. Hahn, U. Hübner-Steiner: Controlled comparative study of Phytodolor N and Amuno® in patients suffering from acute and chronic rheumatic pain conditions. Research Report 04/88

[24] Hahn S., Hübner-Steiner U. (1988). Treatment of painful rheumatic diseases with Phytodolor® in comparison to placebo and Amuno treatment. *Rheumatism, Pain & Inflammation*.

[25] U. Speck, U. Dormehl, M. Bernhardt, A. Keimel, et al.: Comparison of the efficacy and tolerability of Phytodolor® N at various concentrations with diseases of rheumatic origin. Research Report 30/90

[26] M. Bernhardt, A. Keimel, G. Belucci, P. Spasojevic: Double-blind, randomised comparative study of Phytodolor N and placebo as well as an open comparison with Feldene 20 tabs involving convalescent inpatients with arthrotic joint alterations. Research Report 16/91

[27] H. Müller-Faßbender: Comparative investigation of the efficacy of Phytodolor N versus Extractum Populus tremula versus placebo in patients suffering from activated osteoarthritis of the knee. Research Report PFK CR III-004-92 G of June1994

[28] F. Schreckenberger: Pilot study-phytodolor N and Diclofenac for the treatment of hospitalized patients with lateral epicondylitis. Research Report of 1986

[29] F. Schreckenberger: Phytodolor—double-blind trial with placebo and single-blind trial with Diclofenac—three group-comparison. Research Report of August 1987

[30] Schreckenberger F. (1988). The treatment of epicondylitis with Phytodolor. *Der Praktische Arzt*.

[31] W. Schadler, Kalmbach: half-year tolerability of phytodolor N versus diclofenac. Research Report of 1986

[32] D. Baumann, G. Focke, D. Kornasoff: Phytodolor in patients with activated gonarthritis, coxarthritis or shoulder-hand-syndrome—multicentre randomised double-blind study versus diclofenac-sodium. Research Report of November 1989

[33] U. Herzog, J. Fitzek, H. Franek: Phytodolor® N versus Diclofenac: efficacy and tolerance of Phytodolor N solution in comparison with Diclofenac coated tablets in activated arthrosis. Research Report of January 1991

[34] R. Hawel, U. Kinigadner, U. Schmidt: Phytodolor® N and Diclofenac-sodium in the treatment of shoulder-arm-syndrome. Research Report of 1991

[35] R. Hawel, U. Kinigadner, U. Schmidt: Phytodolor® N and Diclofenac-sodium for shoulder-arm syndrome. Poster presentation: Congr German Soc for Phytotherapy. 03.-06.10.1991 in Lübeck-Travemünde/Germany

[36] R. Hawel, U. Kinigadner, H. Gerschpacher, F. Mayrhofer, et al.: Phytodolor versus Diclofenac in rheumatic diseases—controlled randomized double-blind study. Research Report of 1992

[37] J. Michael, H. Sörensen: Comparison of efficacy and tolerance of Phytodolor N and Populus extract in diseases of rheumatic origin. Research Report 31/90

[38] U. Botzenhardt: Phytodolor N—comparative clinical trial against Indomethacin in patients suffering from rheumatoid arthritis. Research Report of 1986

[39] A. Vajda, M. Kiss-Antal: Phytodolor for application in iontophoresis-comparative clinical trial against physiological saline solution. Research Report of June 1990

[40] Ulrich-Merzenich G., Jobst D., Zeitler H., Müller J., Vetter H. (2007). Measurement of synergistic effects of a phytopharmaceutical by microarray-analysis. *Planta Medica*.

[41] Ulrich-Merzenich G., Hartbrod F., Jobst D., Zeitler H. (2008). *Immunomodulation by the Multiextract Mixture Phytodolor® in Fibroblasts*.

[42] Rohnert U., Koske D., Schneider W., Elstner E. F. (1998). Inhibition by salix-extracts and PhytodolorR of copper-catalyzed oxidative destructions. *Zeitschrift für Naturforschung C*.

[43] K.-J. Gundermann: Clinical expert report of February 2012

[44] G. Gotschy, R. Paczenskyv: Multicentre clinical trial (in a private medical practice) of Phytodolor N for patients with predominantly degenerative rheumatic diseases. Research Report of December 1986

[45] T. Krstulovic: Efficacy and tolerability of Phytodolor for patients with rheumatic pain. Research Report of 1981

[46] A. Dunky, R. Eberl: Phytodolor N for the treatment of rheumatic diseases. Research Report of July 1985

[47] Dunky A., Eberl R. (1986). Treatment of rheumatic diseases with a plant-based antirheumatic. *Therapiewoche*.

[48] H. Müller-Faßbender: Phytodolor-saving of prednisolone in patients suffering from rheumatoid arthritis. Research Report of October 1989

[49] F. Rainer, M. Härtel: Phytodolor for Bechterew’s disease—sparing of non-steroidal antirheumatics. Pilot study. Research Report of 1988

[50] Vinson B. (2000). No interactions of the antirheumatic herbal preparation Phytodolor® N∗ with sulfonylureas. *Phytomedicine*.

[51] A. Lahme: 19 therapeutic reports on Phytodolor N for the treatment of fibromyalgia. Therapeutic Report of 1993

[52] H. J. Suschke: Phytodolor N for the treatment of inflammatory joint diseases of every kind of genesis in children 2-16 years of age. Research Report of July 1988

[53] M. Haertel: Phytodolor—clinical trial on the efficacy and tolerability involving 1827 patients. Research Report of 1989

[54] G. Rauch, S. Rauch: Multicentre private practice study of Phytodolor N for diseases of rheumatic origin. Research Report of May 1990

[55] Rauch G., Rauch S. (1991). Rheumatic pain—ash, American aspen (trembling poplar) and golden rod chase it away. Plant-based compound preparation without adverse effects helps in up to 90% of cases. *Ärztl Praxis*.

[56] Adler M., Müller J., Kelber O., Okpanyi S. N., Weiser D. (2009). Retrolektive studie zum einsatz von STW 1 bei erkrankungen des bewegungsapparates in der Praxis. *Zeitschrift für Phytotherapie*.

[57] Ernst E. (1999). The efficacy of Phytodolor for the treatment of musculoskeletal pain—a systematic review of randomised clinical trials. *Journal of Natural Medicines*.

[58] Long L., Soeken K., Ernst E. (2001). Herbal medicines for the treatment of osteoarthritis: a systematic review. *Rheumatology*.

[59] E. Godehardt, M. Ulbrich, Summarised Evaluation of Randomised Double- and Single-Blind Studies with PHYTODOLOR N. Biometric Report of November 2001

[60] Uehleke B., Brignoli R., Rostock M., Saller R., Melzer J. (2011). Phytodolor in musculoskeletal disorders: re-analysis and meta-analysis. *Forschende Komplementärmedizin/Research in Complementary Medicine*.

[61] J. Müller, M. Marin-Galiano, Analysis of effectiveness of Phytodolor vs. non-steroidal anti-inflammatory drugs in the treatment of rheumatic symptoms. Research Report of May 2018

[62] K.-J. Gundermann: Pharmacological-toxicological expert report of February 2012

[63] D. Baumann, H. Frerick, U. Schmidt, N. Schenk: Treatment of acute and subacute soft tissue rheumatism with a plant-based antirheumatic agent. Open study in a private orthopaedic medical practice. Research Report of July 1988

[64] W. Reiter, H. Frerick, U. Schmidt, N. Schenk: Long-term experience with a plant-based antirheumatic for the treatment of chronic polyarthritis. Open 12-week study in a private rheumatological medical practice. Research Report of August 1988

[65] Reiter W., Frerick H., Schmidt U., Schenk N. (1989). What does an antirheumatic agent composed of plant ingredients achieve in the therapy of chronic polyarthritis?. *Therapiewoche*.

[66] S. Speders: Phytodolor for the treatment of degenerative spinal diseases in elderly patients. Research Report of February 1988

[67] Speders S. (1988). Phytodolor® for the treatment of degenerative spinal diseases. *Forum Dr Med*.

[68] Ernst E., Chrubasik S. (2000). Phyto-anti-inflammatories. *Rheumatic Disease Clinics of North America*.

[69] Ernst E. (2008). Complementary treatments in rheumatic diseases. *Rheumatic Disease Clinics of North America*.

[70] Soeken K. L. (2004). Selected CAM therapies for arthritis-related pain: the evidence from systematic reviews. *The Clinical Journal of Pain*.

[71] Access Economics Report (2010). Cost effectiveness of complementary medicines—Phytodolor™ for the treatment of osteoarthritis. *Access Economy Pty Limited*.

